# Unveiling the Potential of Solar-Powered Multistage Hollow Fiber WGMD: A Transient Performance Evaluation

**DOI:** 10.3390/membranes15100318

**Published:** 2025-10-16

**Authors:** Mohamed O. Elbessomy, Kareem W. Farghaly, Osama A. Elsamni, Samy M. Elsherbiny, Ahmed Rezk, Mahmoud B. Elsheniti

**Affiliations:** 1 Mechanical Engineering Department, Faculty of Engineering, Alexandria University, El-Chatby, Alexandria 21544, Egypt; mohamed.elbessomy@alexu.edu.eg (M.O.E.); kareem-wagdy@alexu.edu.eg (K.W.F.); elsamni@alexu.edu.eg (O.A.E.);; 2 Mechanical, Biomedical and Design Engineering Department (MBDE), College of Engineering and Physical Science, Aston University, Birmingham B4 7ET, UK; a.rezk@aston.ac.uk; 3 Energy and Bioproducts Research Institute (EBRI), College of Engineering and Physical Science, Aston University, Birmingham B4 7ET, UK; 4 Mechanical Engineering Department, College of Engineering, King Saud University, Riyadh 11451, Saudi Arabia

**Keywords:** solar membrane distillation, heat recovery, multistage configuration, hollow fiber, water gap

## Abstract

Solar-energy-driven membrane distillation provides a sustainable pathway to mitigate freshwater scarcity by utilizing an abundant renewable heat source. This study develops a two-dimensional axisymmetric computational fluid dynamics (CFD) model to simulate the transient performance of a hollow fiber water gap membrane distillation (HF-WGMD) module integrated with flat-plate solar collectors (FPCs). A lumped-parameter transient FPC model is coupled with the CFD framework to predict feed water temperature under time-varying solar irradiation, evaluated across four representative days in a Mediterranean city. The model is validated against experimental data, showing strong agreement. A comprehensive parametric analysis reveals that increasing the collector area from 10 to 50 m^2^ enhances the average water flux by a factor of 6.4, reaching 10.9 kg/(m^2^h), while other parameters such as collector width, tube number and working fluid flow rate exert comparatively minor effects. The module flux strongly correlates with solar intensity, achieving a maximum instantaneous value of 18.4 kg/(m^2^h) with 35 m^2^ collectors. Multistage HF-WGMD configurations are further investigated, demonstrating substantial reductions in solar energy demand due to internal thermal recovery by the cooling stream. A 40-stage system operating with only 10 m^2^ of solar collectors achieves an average specific thermal energy consumption of 424 kWh/m^3^, while the overall solar desalination efficiency improves dramatically from 2.6% for a single-stage system with 50 m^2^ collectors to 57.5% for the multistage configuration. The proposed system achieves a maximum freshwater productivity of 51.5 kg/day, highlighting the viability and optimization potential of solar-driven HF-WGMD desalination.

## 1. Introduction

Solar energy is one of the most abundant and readily available renewable energy sources, with solar radiation being effectively harnessed to power various sustainable systems. Solar thermal energy can be absorbed and converted into electricity via photovoltaic (PV) panels [1,2], facilitating the operation of electrical devices. Alternatively, it can be directly utilized for thermal applications through different solar collector technologies. Flat plate collectors (FPCs) [3] and evacuated tube collectors (ETCs) [4] are commonly employed for low to medium temperature applications, whereas parabolic trough collectors (PTCs) [5] are utilized for high temperature applications.

Among the various applications of solar energy, solar desalination is regarded as one of the most efficient and sustainable methods for freshwater production. Solar desalination systems are generally classified into two main categories: direct and indirect desalination [6,7,8]. In direct solar desalination, both the evaporation and condensation processes occur within a single integrated unit, as seen in solar stills [9,10] and humidification–dehumidification (HDH) [11] desalination systems. Conversely, the indirect solar desalination approach separates the solar energy collection unit from the desalination process. In this method, solar energy is either harvested using PV panels to power electrically or mechanically driven desalination technologies, such as electrodialysis (ED) [12] and reverse osmosis (RO) [13], or it is captured through various solar collectors to drive thermally powered desalination technologies, including multi-stage flash (MSF) [14,15] and multi-effect distillation (MED) [16].

In addition to these solar desalination technologies, membrane distillation (MD) has emerged as a promising thermally driven, membrane-based desalination technique with significant potential for solar energy integration. MD operates by utilizing a hydrophobic membrane that permits the transport of water vapor while preventing the passage of liquid water. A key advantage of MD is its ability to operate with feed water temperatures below the boiling point, typically in the range of 40–90 °C, making it highly compatible with solar thermal energy sources [17]. This characteristic enhances its feasibility for sustainable and energy-efficient freshwater production, particularly in regions with high solar irradiance. MD is available in several configurations, differentiated by the method of vapor collection and condensation within the permeate side. The four primary configurations include direct contact membrane distillation (DCMD) [18], air gap membrane distillation (AGMD) [19], where water vapor is condensed within the permeate channel, sweeping gas membrane distillation (SGMD) [20], and vacuum membrane distillation (VMD) [21], where the vapor is transported out of the module and condensed externally.

A recent advancement in MD technology is the water gap membrane distillation (WGMD) configuration [22,23,24,25,26], which modifies the AGMD design by filling the air gap with stagnant distillate water. This approach enhances the overall productivity of the module compared to AGMD. Unlike DCMD, the stagnant distillate layer in WGMD eliminates convective heat transfer on the permeate side, thereby reducing conductive heat loss and improving thermal performance [27]. Additionally, the separation of the permeate and cooling streams in WGMD allows for the use of saline water as a coolant, enabling partial recovery of thermal energy from the feed stream prior to main heating [28]. Consequently, WGMD combines the high-water productivity characteristic of DCMD with the improved thermal efficiency of AGMD, offering a promising alternative for efficient solar-driven desalination.

Membrane distillation integrated with direct solar heating has recently emerged in various configurations as a compact and cost-effective desalination approach [29]. Despite its potential, further enhancements are needed to significantly boost productivity and achieve sustainable operation. Elrakhawi et al. [30] developed a one-dimensional finite difference model to analyze a novel sweeping air nanophotonic MD module. The system employed a nanophotonic photothermal coating on a hydrophobic membrane for efficient solar absorption and localized heating, with water vapor transported by a sweeping air stream and condensed on a cooling plate separating the permeate and cooling channels. Performance was tested outdoors in Houston, Texas (July 2019) with a 21,960 ppm saline feed, achieving a distillate flux of 0.65 ± 0.28 kg/(m^2^h) under 784.4 ± 217 W/m^2^ irradiance and 39 °C feed inlet temperature. Model predictions showed that concentrating solar irradiance by a factor of 7.5 increased productivity ninefold, while increasing feed channel thickness from 0.5 to 7.5 mm improved flux by 7%. Meo et al. [31] conducted a systematic evaluation of a direct solar absorption DCMD module using an analytical model, where distributed solar irradiation was directly absorbed on the feed side to heat saline water of 35,000 ppm. The study investigated module performance under various flow configurations and operating conditions. For a counter-current feed and permeate configuration at a feed inlet temperature of 30 °C, Reynolds number of 56.8, and module length of 0.1 m, the permeate flux remained constant at approximately 0.58 kg/(m^2^h) across all solar intensities. When the module length was increased to 1 m under the same conditions, the flux increased from 0.21 kg/(m^2^h) (no solar input) to 0.44 kg/(m^2^h) at 2000 W/m^2^, achieving a 109.5% enhancement. The maximum flux of 0.9 kg/(m^2^h) was observed at a higher feed Reynolds number of 567.7 and 0.1 m module length, but it declined to 0.55 kg/(m^2^h) at 1 m length regardless of solar intensity for both lengths.

Conversely, several studies have investigated the transient performance of various membrane distillation configurations driving by indirect heating using external solar thermal collectors. In these systems, solar collectors are employed to absorb incident solar radiation and supply thermal energy to heat the MD feed water [32,33,34]. Duong et al. [35] developed a one-dimensional empirical model to simulate the performance of a solar-driven DCMD system integrated with an FPC of 22.6 m^2^ for seawater desalination. The DCMD module featured an effective membrane area of 7.2 m^2^. The system operated under transient solar irradiation in New South Wales, Australia, pending 1–5 January 1991. During that period, a maximum water flux of 2.7 kg/(m^2^h) was recorded at a peak feed temperature of 52 °C and a feed flow rate of 900 kg/h. The average daily water production was 142 kg/day, equivalent to 6.3 kg/day per m^2^ of the collector area and 19.7 kg/day per m^2^ of the membrane area. Ma et al. [36] theoretically analyzed the performance of a compact solar-powered VMD system for seawater desalination. The system was driven by solar irradiation, varying from 70 to 640 W/m^2^, over a 12 h period on 1 August in Toulouse, France, using an FPC. A freshwater yield of 3.7 kg/day was achieved with an FPC area of 0.18 m^2^, which increased to 96 kg/day with a collector area of 3 m^2^.

Furthermore, extensive research has been dedicated to solar-powered AGMD systems due to their high thermal efficiency and potential for thermal energy recovery [37,38,39,40]. Sandid et al. [41] conducted both experimental and theoretical studies on a spiral-wound AGMD module. The experiments were carried out with a membrane area of 14.4 m^2^, powered by FPC field with an area of 12 m^2^ side by side with ETC field with an area of 8 m^2^ to desalinate seawater in Port Said, Egypt, on 28th and 29th of June 2020. Maximum water fluxes of 0.9 kg/(m^2^h) and 1.25 kg/(m^2^h) were achieved using FPC and ETC, respectively, at a maximum feed inlet temperature of 91 °C in June. In winter (December), at a reduced feed temperature of 70 °C, fluxes declined by 38.3% (FPC) and 44.4% (ETC). The AGMD module coupled with FPC produced 50 kg/day of freshwater in June, which increased by 57.7% when integrated with ETC. On the other hand, they used mathematical modeling to predict accumulated distillate throughout a complete year. Ruiz-Aguirre et al. [42] conducted pilot-scale experiments on multi-channel spiral-wound AGMD modules (7.2 m^2^ and 24 m^2^ membrane areas) integrated with FPCs delivering up to 7 kW thermal power. They also developed an optimization model, validated experimentally, that could produce 2.44 kg/(m^2^h) of freshwater with a specific energy consumption of 141.5 kWh/m^3^ at 80 °C feed and 20 °C coolant inlet temperatures with a length of 3.72 m and 17.9 m^2^ membrane area.

In contrast, there is a notable scarcity of research on WGMD systems integrated with solar thermal energy [43,44]. Alquraish et al. [44] carried out experimental investigations using a pilot-scale spiral-wound WGMD module with a membrane surface area of 10 m^2^, powered by a solar thermal system comprising a total collector area of 2 m^2^. The experiments were conducted under the climatic conditions of Kairouan, Tunisia, over several days in August 2020. The results demonstrated a maximum freshwater production rate of 15.92 kg/day per m^2^ of membrane surface on August 9th, corresponding to a peak solar irradiance of 978.3 W/m^2^ and a maximum feed inlet temperature of 71.9 °C. Furthermore, the system’s specific thermal energy consumption (STEC) varied between 90 and 310 kWh/m^3^.

The literature reveals a significant gap in studies addressing the performance of WGMD driven by solar energy, particularly for hollow fiber (HF) module configurations, which offer high compactness and scalability. To address this, the present work develops a computational fluid dynamics (CFD) model to simulate the transient performance of an HF-WGMD system integrated with a flat-plate solar collector system. A lumped-parameter transient model of the FPC is coupled with the CFD model to predict the temporal variation in feed water temperature based on the absorbed solar irradiation. The CFD model is validated against experimental data, showing strong agreement.

The system performance is evaluated over four representative days in March 2025 in Alexandria, Egypt, under varying solar intensities. A parametric analysis is conducted to assess the influence of key solar collector parameters, including total collectors’ area, overall width, number of solar tubes and mass flow rate of the working fluid, on the instantaneous and average water flux of the HF-WGMD module. Additionally, the impact of meteorological conditions (solar irradiation and ambient temperature) during the four days on feed inlet temperature and module productivity is examined. Finally, the study investigates multistage HF-WGMD configurations, analyzing their effects on collector area requirements, thermal energy recovery, energy consumption, overall solar desalination efficiency and freshwater productivity.

## 2. Solar-Powered HF-WGMD System: Configuration and Operation

### 2.1. WGMD Module Configuration

**Figure 1:**
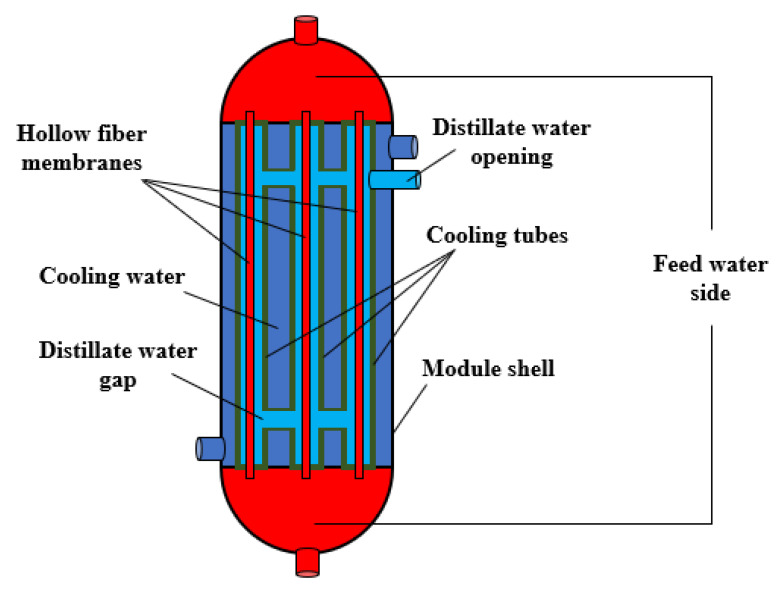
A schematic diagram of HF-WGMD module.

In the present study, a water gap membrane distillation module utilizing hollow fiber membranes is proposed in a shell-and-tube configuration, as illustrated in Figure 1. The module comprises 91 hollow fiber membranes fabricated from hydrophobic polyvinylidene fluoride (PVDF). Each hollow fiber is concentrically aligned within an individual high-density polyethylene (HDPE) cooling tube. All tubes are uniformly arranged within a cylindrical shell of 50 mm inner diameter, with an effective module length of 300 mm. These geometrical specifications were previously optimized by the authors, as reported in Elbessomy et al. [22].

During operation, hot saline feedwater flows through the lumen side of the hollow fiber membranes, while the cooling water is circulated through the shell side of the module in a counter-current flow configuration. The annular spaces between the hollow fibers and their corresponding cooling tubes are filled with distillate water, forming the water gap region. This water gap serves as the medium for vapor condensation and freshwater accumulation. The produced freshwater is subsequently discharged from the module via an overflow outlet, as shown in Figure 1.

### 2.2. Solar-Powered HF-WGMD System Operation

**Figure 2:**
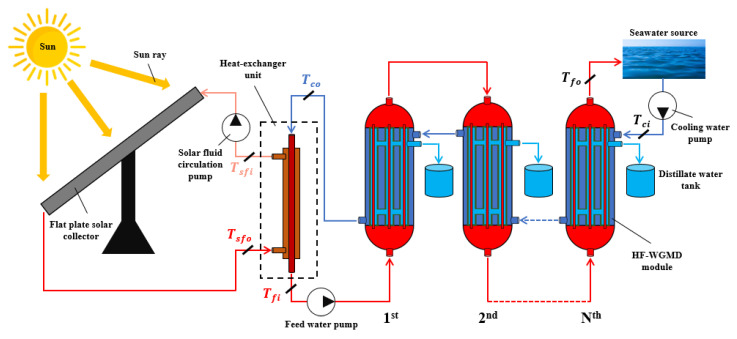
A schematic diagram of the solar-powered multistage HF-WGMD desalination system.

The solar-driven desalination system proposed in this study comprises two primary integrated subsystems: a solar energy harvesting unit and a WGMD desalination unit, thermally linked via a counter-flow heat exchanger, as schematically illustrated in Figure 2.

The solar energy harvesting component utilizes a flat plate solar collector to capture incident solar radiation characteristic of Alexandria, Egypt, throughout four days with different climatic conditions in March 2025. The absorbed solar thermal energy is transferred to a working fluid (water), which circulates through the FPC. This heated solar fluid then passes through the heat exchanger unit, where thermal energy is transferred to the saline feedwater entering the desalination unit.

Simultaneously, seawater is circulated through a series of HF-WGMD modules using a cooling water pump, as depicted in Figure 2. The cooling stream enters the shell side of the modules in reverse sequence, starting from the final module and progressing upstream. This design enables recovery of latent heat from evaporation as well as conductive heat loss from the membrane module feed channels.

After traversing the WGMD modules, the partially heated cooling water is directed through the ideal heat exchanger, where it serves as the primary heating source for the incoming feedwater stream (with the assumption $T_{fi}=T_{sfo}$). The now pre-heated saline water is pumped into the lumens of the hollow fiber membranes, beginning with the first module and advancing sequentially to the last. This counter-current arrangement between the hot feed and cooling streams enhances thermal efficiency and drives effective vapor transport.

During the process, water vapor generated within the feed channels diffuses across the hydrophobic membrane and condenses in the water gap layer, where the distillate is collected. The accumulated freshwater is extracted from each module via an overflow drainage mechanism, ensuring continuous operation and collection of the product water, as shown in Figure 2.

## 3. Theory

A two-dimensional axisymmetric transient model is developed to simulate the desalination process in an HF-WGMD module thermally integrated with a flat-plate solar collector, which serves as the primary heating source for the feed water. In the numerical setup, a single hollow fiber membrane enclosed within a cooling tube is modeled to estimate the permeate flux.

It can be observed that incorporating cooling tubes in the HF-WGMD configuration helps prevent potential interactions between the membrane hollow fibers. However, this aspect requires further experimental investigation to assess the influence of headers and other fittings on the overall module performance. In this study, the predicted performance is scaled according to the total number of hollow fibers in the module to estimate the total water production, assuming uniform performance for all individual hollow fibers enclosed within the cooling tubes.

**Figure 3:**
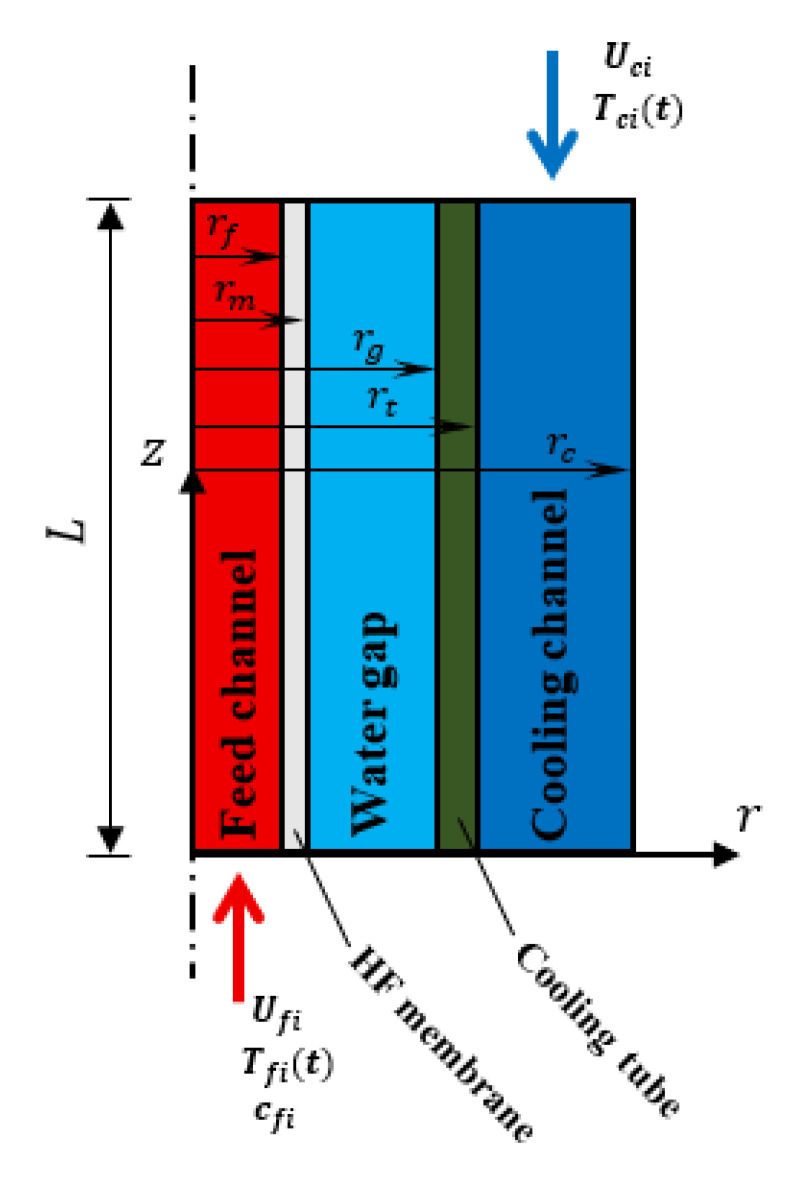
A schematic diagram of the five domains of simulated HF-WGMD module.

The computational domain consists of five concentric, axisymmetric regions arranged radially from the center outward: the feed channel, the hollow fiber membrane, the water gap, the cooling tube wall and the coolant channel, as illustrated in Figure 3. Additionally, the geometrical and operating conditions of HF-WGMD module domain are presented in Table 1.

The model is based on the following assumptions:

- Both feed and coolant flows are laminar.
- The membrane is isotropic in porosity.
- Fouling at the feed-membrane interface is neglected.
- Heat losses to the surroundings are neglected.
- Cooling water temperature is equal to the instantaneous ambient temperature.
- The heat exchanger is ideal and has 100% effectiveness ($T_{fi}=T_{sfo}$).
- The heat loss coefficient of FPC is constant at an average value.

### 3.1. Governing Equations

The two-dimensional cylindrical forms of the transient conservation equations for mass, momentum and energy are solved across the five computational domains of the HF-WGMD module. These equations are simultaneously coupled with the energy conservation equation of the flat-plate solar collector to accurately determine the transient feedwater temperature supplied to the HF-WGMD unit.

#### 3.1.1. Mass Transport Equations

The concentration distributions in both the feed and membrane domains are determined by solving the transient mass transport equations in cylindrical coordinates, as summarized in Table 2. In the feed domain, water vapor transport is governed by the combined effects of convection and diffusion. In contrast, transport across the membrane domain occurs solely via diffusion, in accordance with Fick’s law.

**Table 2:** Transient mass conservation equations of feed and membrane domains in cylindrical coordinates.

Domain	Equation	
Feed	$\frac{\partial c_{f}}{\partial t}+u_{f}\frac{\partial c_{f}}{\partial r}+w_{f}\frac{\partial c_{f}}{\partial z}=\frac{1}{r}\frac{\partial }{\partial r}\left(D_{w}r\frac{\partial c_{f}}{\partial r}\right)+\frac{\partial }{\partial z}\left(D_{w}\frac{\partial c_{f}}{\partial z}\right)$	(1)
Membrane	$\frac{\partial c_{m}}{\partial t}=\frac{1}{r}\frac{\partial }{\partial r}\left(D_{m}r\frac{\partial c_{m}}{\partial r}\right)+\frac{\partial }{\partial z}\left(D_{m}\frac{\partial c_{m}}{\partial z}\right)$	(2)

where $c_{f}$ is the water concentration in feed channel, $u_{f}$ is the feed velocity component in radial direction, $w_{f}$ is the feed velocity component in longitudinal direction, $D_{w}$ is the mutual diffusion coefficient of water and salt, $c_{m}$ is the vapor concentration in membrane layer, $D_{m}$ is the effective membrane diffusion coefficient, t is the time and r and z are the cylindrical coordinates.

The mutual diffusion coefficient of water and salt in the feed solution, $D_{w}$ is estimated using the Wilke–Chang correlation [45], expressed as:

(3) $$D_{w}=\frac{7.4\times 10^{-8}\times {\left(2.6M_{f}\right)}^{1/2}\times {(T}_{f}+273)}{\mu_{f}\times {V_{w}}^{0.6}}$$

where $M_{f}$ is the feed solution molecular mass, $\mu_{f}$ is the feed water dynamic viscosity in cP, and $V_{w}$ is the water molecular volume in cm^3^/mol.

In contrast, mass transport within the membrane domain is governed by a combination of diffusion mechanisms, namely ordinary molecular diffusion and Knudsen diffusion. The relative significance of these mechanisms is determined by evaluating the Knudsen number, which is calculated using the following expression [46]:

(4) $$Kn=\frac{\lambda_{a-v}}{d_{p}}$$

where $d_{p}$ is the average membrane pore diameter and $\lambda_{a-v}$ is the mean free path of vapor molecules, which can be estimated using the following equation:

(5) $$\lambda_{v}=\frac{K_{B}T_{m,avg}}{P\pi {\left[\left(\sigma_{a}+\sigma_{w}\right)/2\right]}^{2}}\times \frac{1}{{\left(1+\left(M_{w}/M_{a}\right)\right)}^{1/2}}$$

where $K_{B}$ is the Boltzmann constant, $T_{m,avg}$ is the average membrane temperature in K, $\sigma_{a}=3.71\;Å$ is the molecular collision diameter of air and $\sigma_{w}=2.64\;Å$ is the molecular collision diameter of water vapor [47].

In the present study, the calculated Knudsen number lies within the transition regime (0.1<Kn<10), confirming that mass transport through the membrane is simultaneously influenced by both molecular diffusion and Knudsen diffusion, with neither mechanism being negligible. Accordingly, the effective mass transport is represented as the combined contribution of these two mechanisms. The corresponding diffusion coefficients for molecular and Knudsen diffusion are evaluated as follows [46]:

(6) $$D_{mol}=1.97\times 10^{-5}{\left(\frac{{(T}_{m}+273)}{256}\right)}^{1.685}$$

(7) $$D_{Kn}=\frac{d_{p}}{3}\times {\left(\frac{8\bar{R}{(T}_{m}+273)}{\pi M_{w}}\right)}^{\frac{1}{2}}$$

While, the effective diffusion coefficient, which accounts for the combined contribution of both mechanisms, is then calculated from:

(8) $$D_{mol-Kn}={\left(\frac{1}{D_{mol}}+\frac{1}{D_{Kn}}\right)}^{-1}$$

To account for the structural characteristics of the membrane, namely porosity and pore tortuosity, the effective membrane diffusion coefficient is expressed as:

(9) $$D_{m}=\frac{\varepsilon }{\tau }D_{mol-Kn}$$

where ε is the membrane porosity and τ is the membrane pore tortuosity which can be estimated by the following formula [48].

(10) $$\tau =\frac{{\left(2-\varepsilon \right)}^{2}}{\varepsilon }$$

#### 3.1.2. Momentum Transport Equations

The continuity and Navier–Stokes equations are solved within the feed and cooling channel domains to obtain the transient pressure and velocity fields. These governing equations are formulated for two-dimensional, incompressible, unsteady, laminar flow in cylindrical coordinates. They are expressed as follows:

(11) $$\frac{1}{r}\frac{\partial (ru)}{\partial r}+\frac{\partial w}{\partial z}=0$$

(12) $$\rho \frac{\partial u}{\partial t}+\rho \left[u\frac{\partial u}{\partial r}+w\frac{\partial w}{\partial z}\right]=-\frac{\partial P}{\partial r}+\mu \left[\frac{1}{r}\frac{\partial }{\partial r}\left(r\frac{\partial u}{\partial r}\right)-\frac{u}{r^{2}}+\frac{\partial^{2}u}{\partial z^{2}}\right]$$

(13) $$\rho \frac{\partial w}{\partial t}+\rho \left[u\frac{\partial u}{\partial r}+w\frac{\partial w}{\partial z}\right]=-\frac{\partial P}{\partial z}+\mu \left[\frac{1}{r}\frac{\partial }{\partial r}\left(r\frac{\partial w}{\partial r}\right)+\frac{\partial^{2}w}{\partial z^{2}}\right]$$

where ρ is density, μ is dynamic viscosity and P is domain local pressure.

#### 3.1.3. Energy Transport Equations

Temperature distribution plays a pivotal role in this study, as it directly governs the local saturation concentrations on both sides of the membrane and influences the temperature-dependent diffusion coefficients. To capture these effects, the energy conservation equation, whose mathematical formulations are summarized in Table 3, is solved simultaneously with the mass and momentum transport equations across all five domains of the HF-WGMD module. In the feed and cooling channel domains, heat transfer occurs through a combination of convection and conduction. In contrast, heat transfer within the membrane, water gap and cooling tube wall domains is restricted to conduction, as also presented in Table 3.

**Table 3:** Transient energy conservation equations of HF-WGMD module domains in cylindrical coordinates.

Domain	Equation	
Feed	$\rho C_{P}\frac{\partial T}{\partial t}+\rho C_{P}\left[u\frac{\partial T}{\partial r}+w\frac{\partial T}{\partial z}\right]=\frac{1}{r}\frac{\partial }{\partial r}\left(kr\frac{\partial T}{\partial r}\right)+\frac{\partial }{\partial z}\left(k\frac{\partial T}{\partial z}\right)$	(14)
Coolant		
Membrane	$\frac{\partial T}{\partial t}=\frac{1}{r}\frac{\partial }{\partial r}\left(kr\frac{\partial T}{\partial r}\right)+\frac{\partial }{\partial z}\left(k\frac{\partial T}{\partial z}\right)$	(15)
Water gap		
Cooling tube		

where $C_{P}$ is specific heat at constant pressure, T is domain local temperature, k is thermal conductivity.

The effective thermal conductivity of the membrane, $k_{m}$, is estimated by accounting for both the solid polymer material and the vapor within the pores. It is expressed as a porosity-weighted combination of the membrane material conductivity and the vapor conductivity, as follows:

(16) $$k_{m}=\varepsilon k_{v}+\left(1-\varepsilon \right)k_{s}$$

(17) $$k_{v}=0.0144-2.16\times 10^{-5}{(T}_{m}+273)+1.32\times 10^{-7}{\left(T_{m}+273\right)}^{2}$$

where $k_{m}$ is the effective thermal conductivity of membrane domain, $k_{v}$ is the water vapor thermal conductivity and $k_{s}$ is the thermal conductivity of solid PVDF membrane material.

#### 3.1.4. FPC Mathematical Model

The flat-plate collector system, comprising an array of parallel and series-connected collectors each with an aperture area of $1\times 1\;m^{2}$, is modeled as a transient lumped-parameter system. The detailed specifications of the FPC system, operating with water as the working solar fluid, are summarized in Table 4.

The width of the collectors’ array corresponds to the number of collectors connected in parallel. For example, an array width of 5 m represents five collectors arranged in parallel. Conversely, the length of the array represents the number of collectors connected in series.

The total area of the array is calculated as the product of its width and length. The number of tubes is counted along the width direction. For instance, in an array that is 5 m wide and 10 m long with a total of 40 tubes, each 1 m-wide collector contains 8 tubes. The total length of the connected tubes in series is therefore 10 m (representing 10 series-connected collectors).

The specific configuration of the collectors will be defined in each results section according to the requirements of the investigation.

The instantaneous useful heat gain by the solar fluid in the FPC, which represents the thermal energy transferred to the saline feed water of the desalination unit, is calculated using the following expression [49]:

(18) $$Q_{u}(t)=F_{R}A_{sc}\left[\tau_{g}\alpha_{p}S_{i}(t)-U_{L}\left(T_{sfi}(t)-T_{amb}(t)\right)\right]$$

where $Q_{u}(t)$ is the instantaneous useful heat gain by the solar fluid, $A_{sc}$ is the FPC area, $T_{sfi}(t)$ is the instantaneous solar fluid inlet temperature, $\tau_{g}$ is the glass transmissivity, $\alpha_{p}$ is the plate absorptivity, $U_{L}$ is the overall heat loss coefficient, $S_{i}(t)$ is the instantaneous solar irradiance on the FPC, $T_{amb}(t)$ is the instantaneous ambient temperature and $F_{R}$ is the heat removal factor which can be calculated using the equation [49]:

(19) $$F_{R}=\frac{{\dot{m}}_{sf}C_{P_{sf}}}{A_{sc}U_{L}}\left[1-e^{-\frac{{U_{L}F}^{\prime }A_{sc}}{{\dot{m}}_{sf}C_{P_{sf}}}}\right]$$

where ${\dot{m}}_{sf}$ is the solar fluid mass flow rate, $C_{P_{sf}}$ is the solar fluid specific heat at constant pressure and $F^{\prime }$ is the collector efficiency factor and expressed as follows [49]:

(20) $$F^{\prime }=\frac{\frac{1}{U_{L}}}{S_{st}\left[\frac{1}{U_{L}(d_{sto}+\left(S_{st}-d_{sto}\right)F}+\frac{1}{C_{b}}+\frac{1}{\pi d_{sti}h_{sf}}\right]}$$

where $d_{sto}$ and $d_{sti}$ are the inside and outside solar tube diameters, respectively, $S_{st}$ is the solar tube spacing in the FPC, $C_{b}$ is the bond conductance, $h_{sfi}$ is the solar fluid convection heat transfer coefficient and F is the standard fin efficiency that can be calculated using the following equation [49]:

(21) $$F=\frac{\tanh\left[\frac{m\left(S_{st}-d_{sto}\right)}{2}\right]}{\frac{m\left(S_{st}-d_{sto}\right)}{2}}$$

(22) $$m=\sqrt{\frac{U_{L}}{k_{p}\delta_{p}}}$$

where $k_{p}$ is the absorber plate thermal conductivity and $\delta_{p}$ is the absorber plate thickness.

On the other hand, the ideal heat exchanger is assumed to transfer the thermal energy gained by the solar fluid directly to the feed water supplied to the HF-WGMD module. Accordingly, the instantaneous outlet temperature of the solar fluid from the FPC, and thus the inlet feed water temperature to the desalination system, is determined as follows:

(23) $$T_{fi}\left(t\right)=T_{sfo}\left(t\right)=\frac{Q_{u}(t)}{{\dot{m}}_{sf}C_{P_{sf}}}+T_{sfi}(t)$$

where $T_{fi}\left(t\right)$ is the instantaneous feed water inlet temperature to the desalination module.

### 3.2. Boundary Conditions

The governing equations described above are coupled with appropriate boundary conditions applied across all computational domains, thereby forming a complete mathematical model for numerical solution. These boundary conditions (BC) specify the physical constraints at the inlets, outlets, walls and the interfaces between adjacent domains. The subsequent sections present the detailed boundary conditions adopted for each of the conservation equations.

#### 3.2.1. Mass Transport BC

Table 5 summarizes the boundary conditions imposed on the mass transport equations within the feed and membrane domains. Specifically, the concentration at the hot feed-membrane interface ($c_{hot}$) and at the cold membrane-water gap interface ($c_{cold}$) are prescribed according to the saturation concentration of water vapor corresponding to the local temperature at each interface.

The vapor concentrations at the hot and cold membrane interfaces are determined from the saturation vapor pressures evaluated at the local membrane-water gap interface temperature, using the standard Antoine equation. At the feed-membrane interface, however, the effect of feed water salinity is taken into account by employing a modified form of the Antoine equation, which reduces the saturation pressure relative to that of pure water [50].

(24) $$P_{sat_{cold}}=133.416\;\times \;10^{8.10765-\frac{1750.286}{T_{m}+235}}$$

(25) $$P_{sat_{hot}}=x_{w}a_{w}\;\times \;133.416\;\times \;10^{8.10765-\frac{1750.286}{T_{m}+235}}$$

where $x_{w}$ denotes the mole fraction of water and $a_{w}$ represents the water activity coefficient. Both $x_{w}$ and $a_{x}$ are calculated using the following equations [51]:

(26) $$x_{w}=1-x_{NaCl}$$

(27) $$a_{w}=1-0.5x_{NaCl}-10x_{NaCl}^{2}$$

where $x_{NaCl}$ is the mole fraction of salt in the feed solution.

These saturation pressures are subsequently employed to calculate the corresponding vapor concentrations at both the feed–membrane and membrane–water gap interfaces, as expressed by the following equations:

(28) $$c_{sat}=\frac{W_{v}}{v_{v}M_{w}}$$

(29) $$W_{v}=0.62\frac{P_{sat}}{P_{atm}-P_{sat}}$$

(30) $$v_{v}=0.29\;{(T}_{m}+273)\frac{1+1.61\;W_{v}}{10^{-3}\;P_{atm}}$$

where $c_{sat}$ is the saturation concentration of water vapor, $T_{m}$ is the membrane local temperature, $v_{v}$ is the specific volume of water vapor, $W_{v}$ is the vapor mass content and $P_{atm}$ is the atmospheric pressure.

#### 3.2.2. Momentum Transport BC

The boundary conditions applied to the momentum transport equations, governing the feed and coolant channel domains, are summarized in Table 6.

#### 3.2.3. Energy Transport BC

The heat energy conservation equation is solved simultaneously across all five domains of the HF-WGMD module. Accordingly, the external boundary conditions governing heat transport in the module are summarized in Table 7.

Additionally, two internal boundary conditions are imposed within the feed channel and water gap domains. At the feed–membrane interface, a boundary heat sink is applied to account for the thermal energy consumed from the feed water during evaporation. Conversely, at the membrane-water gap interface, a boundary heat source is introduced to represent the release of latent heat to the distillate water during vapor condensation. In both cases, the corresponding amounts of latent heat flux are evaluated using the following expression:

(31) $$q_{Latent}=\frac{J}{3600}h_{fg}$$

where J denotes the distillate water flux and $h_{fg}$ represents the latent heat of vaporization (or condensation). Both quantities are calculated using the following relations:

(32) $$J\left(t\right)[\mathrm{kg}/(m^{2}h)]=-D_{m}\frac{\partial c_{m}}{\partial r}{|}_{r=r_{m}}\times M_{w}\times 3.6$$

(33) $$h_{fg}(t)=(2494-2.2\;T_{m})\times 10^{3}$$

where $D_{m}$ is the membrane diffusion coefficient, $c_{m}$ is the local vapor concentration in membrane, $M_{w}$ is the water molecular mass and $T_{m}$ is the membrane local temperature.

### 3.3. Performance Indicators of the Solar Desalination System

The performance of the proposed solar-driven HF-WGMD system is assessed through the evaluation of three key parameters: the specific thermal energy consumption, the overall system efficiency and the freshwater productivity. The instantaneous STEC of the HF-WGMD module is defined as the amount of thermal energy supplied to the feed stream per unit volume of distillate produced. It can be expressed mathematically as:

(34) $$STEC\left(t\right)\;[kWh/m^{3}]=\frac{\rho_{w}\times \dot{m_{f}}\times C_{p_{f}}\times (T_{fi}(t)-T_{co}(t))}{J(t)\times N_{f}\times N_{stages}\times 2\pi r_{m}\times L\times 10^{-3}}$$

(35) $$\dot{m_{f}}\;[kg/s]=\rho_{f}\times \pi {r_{f}}^{2}\times 10^{-6}\times U_{fi}\times N_{f}$$

Here, $\rho_{w}$ is the distillate water density, $\dot{m_{f}}$ is the feed water mass flow rate, $C_{p_{f}}$ is the feed water specific heat at constant pressure, $T_{fi}(t)$ is the instantaneous feed water inlet temperature, $T_{co}(t)$ is the instantaneous cooling water outlet temperature, $N_{f}$ is the number of fibers inside the module, $N_{stages}$ is the number of module stages, $r_{m}$ is the membrane outer radius, L is the module effective length, $r_{f}$ is the feed channer radius, J(t) is the instantaneous distillate water flux and $\rho_{f}$ is the feed water density.

Moreover, the average STEC over the entire daytime operation period is employed to evaluate the overall thermal energy consumption of the HF-WGMD modules. It is determined as:

(36) $$Avg.STEC[kWh/m^{3}]=\frac{\int_{0}^{∆t}STEC\left(t\right)\;dt}{∆t}$$

where ∆t is the daytime operation period.

The solar desalination efficiency of the solar-driven HF-WGMD system is defined as the ratio of the thermal energy effectively utilized for water evaporation at the feed-membrane interface to the total solar energy incident on the flat-plate collector array. Accordingly, the instantaneous system efficiency is expressed as:

(37) ηt%=J(t)3600×Nf×Nstages×2πrm×L×10−6×hfg(t)Si(t)×Asc×100

where $h_{fg}(t)$ is the latent heat of vaporization, $S_{i}(t)$ is the instantaneous solar irradiance on the FPC and $A_{sc}$ is the solar collector area.

The average overall efficiency of the solar-driven HF-WGMD system over the daytime operation period is obtained by integrating the instantaneous efficiency with respect to time and normalizing by the total operation duration. It is expressed as:

(38) $$Avg.\eta \left[\%\right]=\frac{\int_{0}^{∆t}\eta \left(t\right)\;dt}{∆t}$$

On the other hand, different forms of productivity are considered in the present study to evaluate the performance of the proposed multistage HF-WGMD systems. The specific productivity (SP) per unit membrane area is defined as:

(39) $$SP\;[kg/(m_{m}^{2}day)]=\frac{\int_{0}^{∆t}J\left(t\right)\;dt}{∆t}\times 24$$

In contrast, the specific productivity normalized by the solar collector area quantifies the freshwater yield relative to the effective collector surface. It is mathematically expressed as:

(40) $$SP\;[kg/(m_{sc}^{2}day)]=\frac{\int_{0}^{∆t}J\left(t\right)\;dt}{∆t}\times \frac{N_{f}\times N_{stages}\times 2\pi r_{m}\times L\times 10^{-6}}{A_{sc}}\times 24$$

The overall productivity represents the cumulative daily freshwater yield of the desalination system in kg/day and is mathematically expressed as:

(41) $$Productivity=\frac{\int_{0}^{∆t}J\left(t\right)\;dt}{∆t}\times N_{f}\times N_{stages}\times 2\pi r_{m}\times L\times 10^{-6}\times 24$$

### 3.4. Solution Technique

The numerical model couples the governing transport equations with supplementary algebraic relations, solved simultaneously in COMSOL Multiphysics 5.4 using the finite element method. Transient mass, momentum, and heat transport within the HF-WGMD module are resolved using the *Transport of Diluted Species*, *Laminar Flow*, and *Heat Transfer* models. The flat-plate solar collector is represented by a *transient lumped-parameter* model, which is applicable under adaptable *Mathematics* models, where the energy balance equations (Equations (18)–(23)) predict the time-dependent outlet temperature of the solar working fluid based on absorbed solar irradiation, ambient temperature, and collector characteristics. This predicted outlet temperature is directly applied as the inlet boundary condition for the feed stream entering the HF-WGMD module, thereby establishing a coupled simulation framework between the solar collector and CFD models.

Additional user-defined equations are implemented to account for vapor diffusion coefficients (Equations (3) and (6)–(9)), Antoine correlations for vapor pressure (Equations (24) and (25)), and temperature-dependent thermophysical properties. The complete set of equations is solved concurrently under specified initial and boundary conditions, with the distillate side assumed to be salt-free (zero solute concentration).

### 3.5. Grid Independence Study

A grid independence study is conducted to evaluate the influence of mesh density on the accuracy of the numerical solution while minimizing computational cost. Three grid resolutions are tested for the simulated geometry of a 300 mm HF-WGMD module integrated with a solar collector array of 50 m^2^ total area under transient conditions corresponding to solar irradiation and ambient temperature on Day 1. The water flux is evaluated at three different daytime hours (09:00, 12:00 and 15:00) for grids consisting of 112,715, 348,567 and 1,017,025 elements.

**Figure 4:**
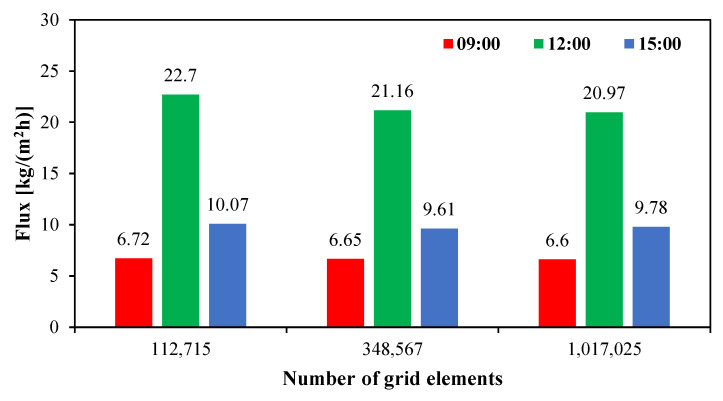
Grid independence study.

The results, presented in Figure 4, indicate that increasing the number of grid elements beyond 348,567 yields no significant improvement in solution accuracy. Specifically, tripling the number of elements from 348,567 to 1,017,025 alters the predicted water flux by only 0.75%, 0.91% and 1.77% at 09:00, 12:00 and 15:00, respectively. Based on these findings, a grid size of 348,567 elements is adopted for the present transient CFD simulations to ensure accurate numerical predictions while minimizing computational time.

## 4. Results and Discussion

### 4.1. Experimental Validation

The validity of the present mathematical model is established by comparing the CFD simulation results with the experimental data reported by Gao et al. [52] for a HF-WGMD module operated under various conditions at a feed inlet salinity of 10,000 ppm. The experimental setup consisted of a shell with an inner diameter of 25 mm, housing eight hollow fibers inserted within eight cooling tubes, with an effective module length of 425 mm. The feed stream was circulated through the lumen of the hollow fibers, while the coolant fluid was introduced on the shell side. The annular gap formed between each fiber and its corresponding cooling tube was filled with stagnant water, serving as the distillate collection region.

**Figure 5:**
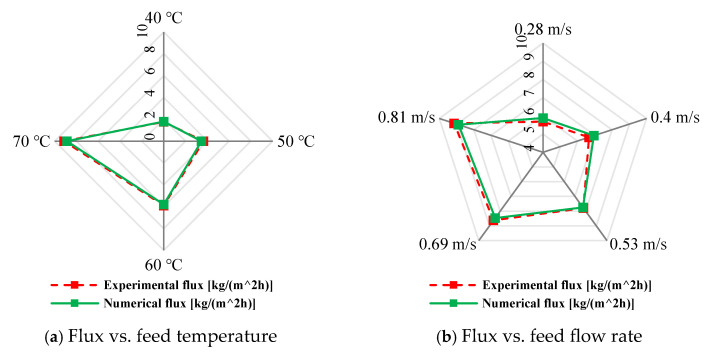
CFD model validation with experimental data of Gao et al. [52].

Overall, the numerical predictions demonstrated close agreement with the experimental measurements. Figure 5a illustrates the effect of feed inlet temperature on water flux at feed and coolant inlet velocities of 0.81 m/s and 0.008 m/s, respectively. The largest deviation occurred at a feed temperature of 50 °C, where the model underpredicted the flux by 4.9%. Similarly, the influence of feed and coolant inlet velocities was examined at a feed temperature of 70 °C, as shown in Figure 5b. At a feed velocity of 0.4 m/s, the model predicted a flux of 6.95 kg/(m^2^h) compared to the experimental value of 6.67 kg/(m^2^h), corresponding to a maximum deviation of +4.22%. In general, the percentage deviations obtained across all operating conditions were within acceptable limits and can be reasonably attributed to the simplifying assumptions inherent in the numerical model.

### 4.2. Solar Irradiance and Ambient Temperature

**Figure 6:**
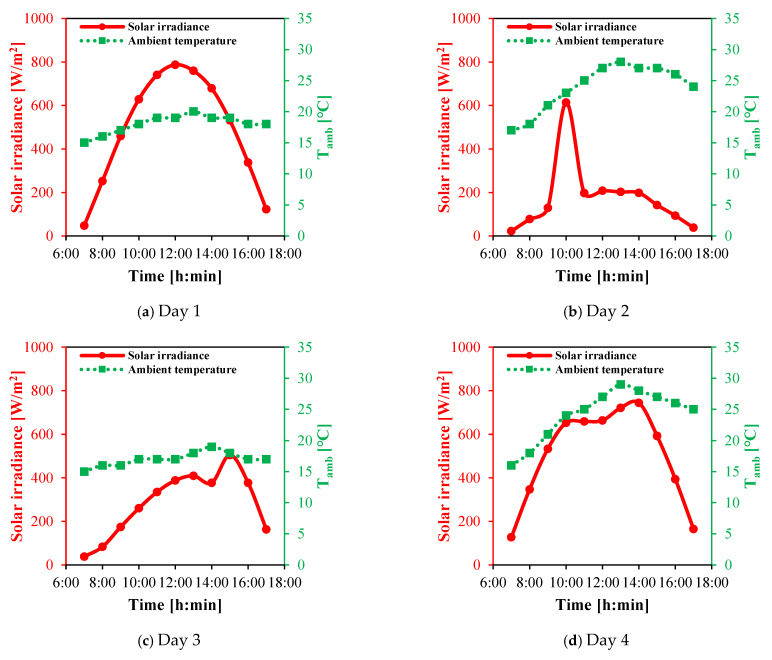
Solar irradiance and ambient temperature during daytime of four days in March 2025 in Alexandria, Egypt.

Solar radiation and ambient temperature data were recorded for four days in Alexandria, Egypt, on March 6th (Day 1), 17th (Day 2), 22nd (Day 3), and 26th (Day 4), 2025, from 07:00 to 17:00, as illustrated in Figure 6. Under clear sky (ideal) conditions of Day 1, the incident solar heat flux exhibited a parabolic profile peaking at 787 W/m^2^ around noon (12:00, as shown in Figure 6a.

In contrast, significantly lower solar irradiance levels were observed under non-ideal sky conditions on Days 2 and 3. On Day 2, the solar heat flux predominantly ranged between 22 and 208 W/m^2^ throughout the day, except for a brief peak of 613 W/m^2^ recorded at 10:00, as shown in Figure 6b. On Day 3, the maximum solar flux reached 505 W/m^2^, as shown in Figure 6c.

On Day 4 (semi-ideal), the solar irradiation gradually increased from 127 W/m^2^ in the early morning to 652 W/m^2^, where it remained nearly constant for approximately two hours. It then continued rising to a peak value of 744 W/m^2^ at 14:00 before declining to 165 W/m^2^ by 17:00, as illustrated in Figure 6d.

In addition to solar flux, ambient temperature variations were also recorded. On Days 1 and 3, ambient temperatures ranged between 15 °C and 20 °C (Figure 6a,c). Higher temperatures were recorded on Days 2 and 4, reaching maximum values of 28 °C and 29 °C, respectively (Figure 6b,d).

### 4.3. Parametric Study on the FPC System

In the following sections, a parametric study is carried out to evaluate the influence of geometrical and operating conditions of flat-plate solar collector array integrated with a HF-WGMD module. The objective is to identify the optimal collector array design that can efficiently supply the membrane distillation unit with the required thermal energy. In this analysis, a single HF-WGMD module is considered, consisting of 91 hollow fibers housed in a 50 mm shell with an effective length of 300 mm. The investigations in this section are under the weather conditions of Day 1.

#### 4.3.1. Solar Collector Overall Surface Area

**Figure 7:**
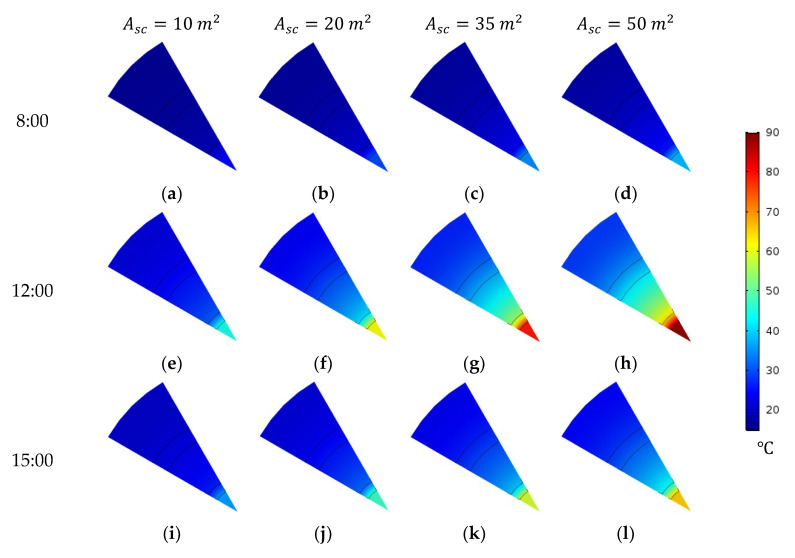
Temperature contours through a cross-sectional plane of the HF-WGMD module at z=0 integrated with different solar collectors’ areas during the operation on Day 1.

**Figure 8:**
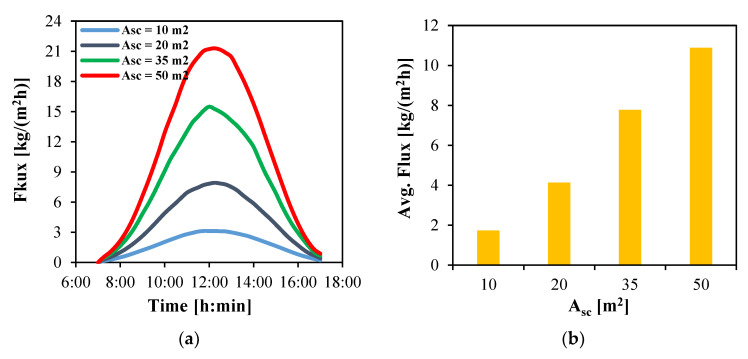
The water flux produced by HF-WGMD module integrated with different solar collectors’ areas during the operation on Day 1; (**a**) instantaneous and (**b**) average.

The influence of the total surface area of the solar collectors on the feed inlet temperature, and consequently on the water production flux of the module, is investigated under clear-sky conditions on Day 1. In this analysis, the overall width of the FPC system is fixed at 5 m, with five rows of collectors connected in parallel and 2, 4, 7 and 10 rows arranged in series, corresponding to total collector areas of 10, 20, 35 and 50 m^2^, respectively. Larger collector areas absorb more thermal energy, leading to higher feed inlet temperatures and increased distillate flux, as shown in Figure 7 and Figure 8.

Figure 7 illustrates temperature contours across the MD module’s cross-section at z=0 at different times, highlighting the impact of solar collector system size. At 12:00, when solar irradiance reaches its peak, the feed inlet temperature increases significantly with larger collector array. Specifically, enlarging the total solar collector area from 10 m^2^ to 20, 35 and 50 m^2^ leads to notable temperature rises, as shown in Figure 7e–h. The average feed inlet temperature correspondingly increases from 44.8 °C to 61.4, 78.9 and 88.9 °C, respectively, as summarized in Table 8. When the total collector area is fixed (e.g., at 50 m^2^), the feed inlet temperature exhibits a strong temporal dependence due to variations in solar irradiance over the course of the day.

The instantaneous water vapor flux produced by the MD module is illustrated in Figure 8a. The maximum flux is observed at 12:00 for all collector array configurations, coinciding with the highest incident solar radiation. The maximum flux reaches 21.2 kg/(m^2^h) with a total area of 50 m^2^. This value decreases substantially by approximately 26.9%, 63.2% and 85.4% when the area is reduced to 35, 20 and 10 m^2^, respectively. The flux also varies significantly with time.

The average water flux over the 10 h daytime period is presented in Figure 8b. It increases proportionally with the total collector area. Larger collector areas capture more thermal energy, which raises the feed water temperature and consequently enhances the module’s productivity. The highest average flux of 10.9 kg/(m^2^h) is obtained using a 50 m^2^ solar collector array. In comparison, average fluxes of 7.8, 4.1 and 1.7 kg/(m^2^h) are achieved for areas of 35, 20 and 10 m^2^, respectively.

#### 4.3.2. Solar Collector Overall Width

**Figure 9:**
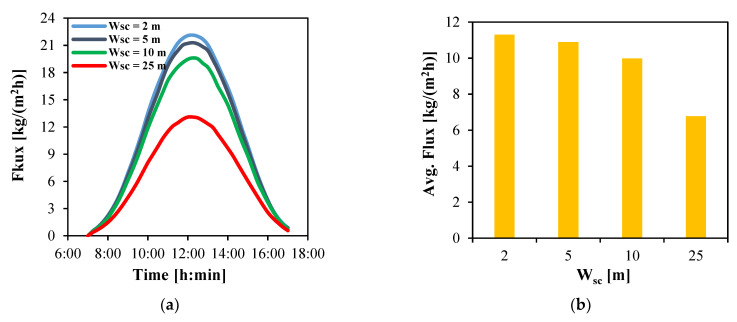
The water flux produced by HF-WGMD module integrated with different solar collectors’ overall widths during the operation on Day 1; (**a**) instantaneous and (**b**) average.

A solar collector array with a total area of 50 m^2^ comprising 60 tubes per row is examined in this section to evaluate the influence of collector array geometry, specifically its total width and length, on the performance of the MD module. In this analysis, the total width is systematically varied from 2 m to a maximum of 25 m, with the corresponding collectors’ total length adjusted inversely from 25 m to 2 m in order to maintain a constant surface area. It should be noted that when the same mass flow rate and the same number of tubes are used, an array with a width of 5 m and a length of 10 m results in a longer residence time of the solar working fluid within the collector array compared to an array with a width of 10 m and a length of 5 m. Consequently, the working fluid attains a higher temperature in configurations with a longer length (and shorter width) and the MD modules exhibit enhanced water production flux, as demonstrated in Figure 9.

Figure 9a presents the temporal evolution of the instantaneous water flux for MD modules connected to solar collector arrays of varying total widths. In all cases, the flux increases with time, peaking at 12:00 (corresponding to the highest solar irradiance observed on Day 1 and hence highest feed inlet temperature) before gradually decreasing toward the end at 17:00. Smaller array widths consistently produce higher fluxes. For example, at 12:00, a 2 m-wide array achieves a maximum flux of 22.1 kg/(m^2^h), compared to 21.2 kg/(m^2^h) for 5 m, 19.5 kg/(m^2^h) for 10 m, and 13.1 kg/(m^2^h) for 25 m widths.

The average water flux over the 10 h daytime operation is also evaluated for each configuration, as depicted in Figure 9b. The results indicate that the system incorporating a 2 m-wide collector array produces an average flux of 11.3 kg/(m^2^h), which decreases slightly to 10.9 and 10 kg/(m^2^h) for widths of 5 and 10 m, respectively. In contrast, the average flux drops markedly to 6.8 kg/(m^2^h) for the configuration with a 25 m-wide collector array.

#### 4.3.3. Total Number of Collector Tubes

**Figure 10:**
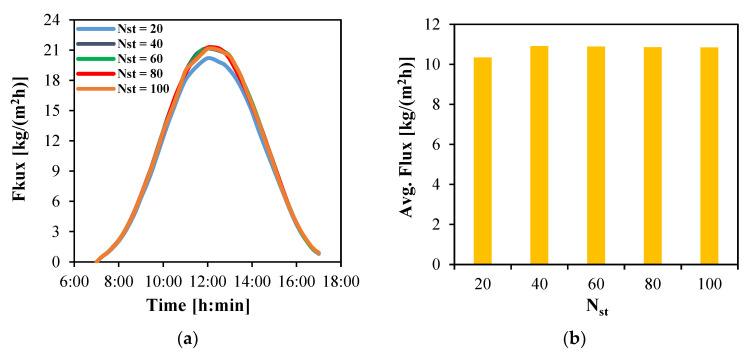
The water flux produced by HF-WGMD module integrated with different solar collectors’ tube counts during the operation on Day 1; (**a**) instantaneous and (**b**) average.

In this section, the performance of the MD module is analyzed when integrated with a solar collector array of fixed total area (50 m^2^) and constant width (5 m), arranged as five parallel rows and ten series rows. The analysis examines the influence of varying the number of collector tubes, ranging from 20 to 100. At lower number of tubes up to 40, increasing the number of tubes decreases the spacing between them, thereby enhancing optical concentration and thermal collection efficiency through improved collector removal factor. As a result, the absorbed solar energy is increased. Conversely, a greater number of tubes raises the working fluid mass flow rate, which increases the overall heat capacity of the circulating fluid and consequently lowers the outlet temperature. The balance between these competing effects governs the resulting trends in water production flux, as illustrated in Figure 10.

As presented in Figure 10a, all collector configurations exhibit a similar temporal flux profile, with the water production flux peaking at 12:00. The average water flux over the full 10 h daytime operation period is shown in Figure 10b. The results indicate that configurations with more than 20 tubes achieve an average flux of up to 10.9 kg/(m^2^h), whereas the 20-tube configuration yields a marginally lower average flux of approximately 10.3 kg/(m^2^h).

#### 4.3.4. Solar Fluid Mass Flow Rate

The effect of the solar fluid mass flow rate on the performance of the MD module is investigated by simulating a system driven by a 50 m^2^ solar collector array with a width of 5 m and comprising 40 tubes per row. Increasing the fluid flow rate through the collectors reduces residence time and the thermal energy gained per unit of fluid, resulting in lower outlet temperatures. This lower temperature reduces water production of the MD membrane.

**Figure 11:**
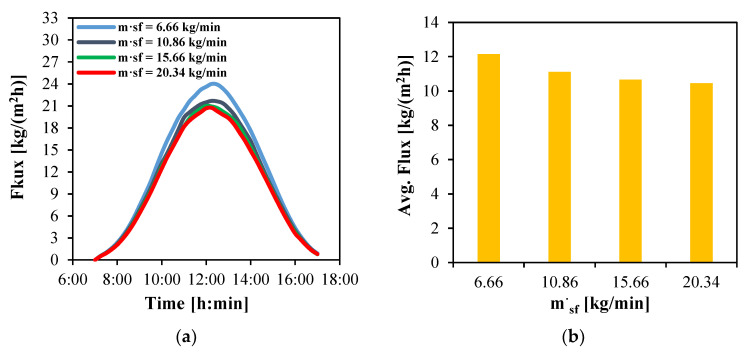
The water flux produced by HF-WGMD module integrated with different solar collectors’ mass flow rates during the operation on Day 1; (**a**) instantaneous and (**b**) average.

Figure 11 illustrates the influence of various solar fluid mass flow rates on both the instantaneous and average water flux. As shown in Figure 11a, all tested flow rates exhibit a similar parabolic trend in instantaneous flux, peaking around solar noon (12:00). The lowest flow rate of 6.66 kg/min consistently outperforms the higher rates, achieving a maximum water flux of 23.6 kg/(m^2^h). In contrast, increasing the flow rate to 10.86, 15.66 and 20.34 kg/min reduces the peak flux to 21.5, 21 and 20.7 kg/(m^2^h).

The corresponding average water flux over the 10 h daytime operation is presented in Figure 11b. The highest average flux of 12.1 kg/(m^2^h) is obtained at the lowest flow rate (6.66 kg/min). Increasing the flow rate leads to progressively lower average fluxes, with reductions of approximately 8.3%, 11.6% and 13.2% for flow rates of 10.86, 15.66 and 20.34 kg/min, respectively.

### 4.4. The Effect of Weather Conditions at Different Days

The performance of a HF-WGMD module integrated with a flat plate solar collector array was evaluated under variable weather conditions during March 2025 in Alexandria, Egypt. The membrane module was designed with 91 hollow fibers housed within a shell of 50 mm diameter and an effective fiber length of 300 mm. To supply the required thermal energy, the module was coupled with a solar collector system comprising a 35 m^2^ total collecting area (5 rows in parallel and 7 rows in series) and 40 tubes.

#### 4.4.1. Module Temperatures

**Figure 12:**
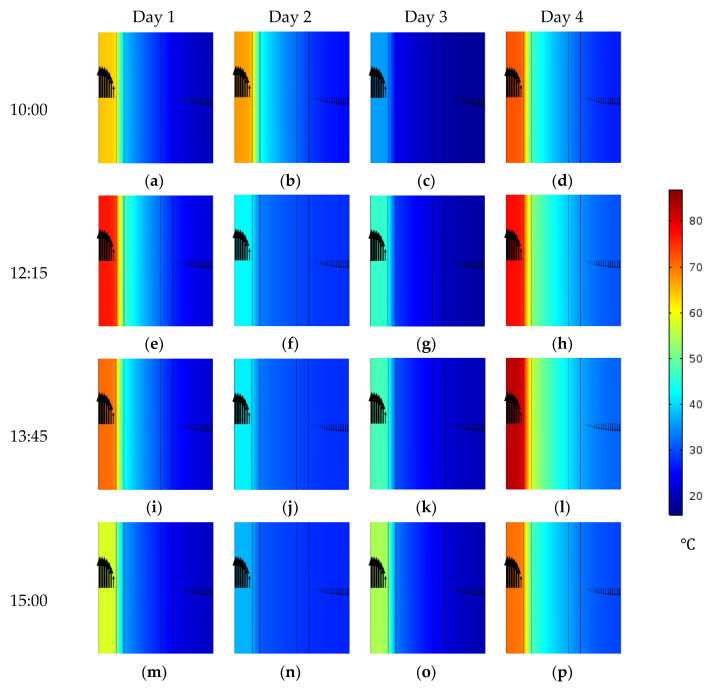
Temperature contours in a 3 mm section in the middle of HF-WGMD module during daytime operation of four days in March 2025.

Figure 12 presents CFD visualizations of a middle section of the module, illustrating the temperature distribution across the five domains at different hours over the four days. The results indicate that the timing of the highest feed water temperature varies from day to day, coinciding with the peak solar flux reported in Figure 6. Furthermore, the cooling channels exhibit lower temperatures on Day 1 and Day 3 compared to Day 2 and Day 4, a trend that aligns with the corresponding ambient temperature data shown in Figure 6. Notably, the feed channel reaches its highest temperatures on Day 4 at all considered hours, compared with the other days.

Since module productivity is strongly influenced by the hot-side membrane temperature, the average temperature at the feed-membrane interface is examined and summarized in Table 9. On Day 1, the interface temperature peaks at 73.2 °C at 12:15, whereas lower values of 41.8 °C and 44.8 °C are observed on Day 2 and Day 3, respectively, at the same time. A slight increase is recorded on Day 4, where the temperature reaches 74.3 °C. On Day 2, the maximum temperature of 64.6 °C occurs earlier at 10:00, followed by a steady decline to 37 °C at 15:00. The lowest daily maximum is observed on Day 3, with an interface temperature of 52.6 °C at 15:00. In contrast, the highest value across all days is recorded on Day 4, where the interface temperature reaches 79.1 °C at 13:45.

#### 4.4.2. Module Flux

**Figure 13:**
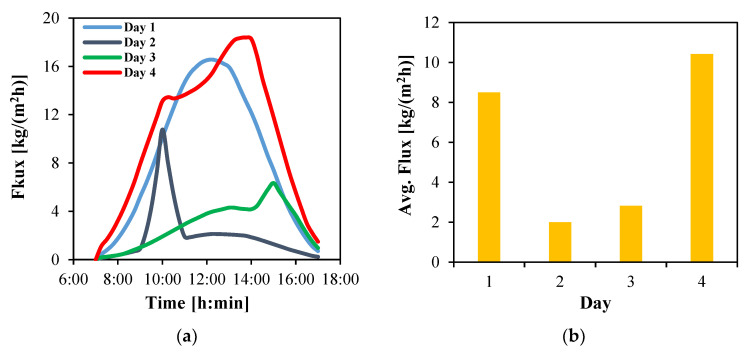
The water flux produced by HF-WGMD module integrated with a 35 m^2^ solar collector array during daytime operation of four days in March 2025; (**a**) instantaneous and (**b**) average.

The instantaneous water flux is presented in Figure 13a for the four days. The flux variations closely follow the trends of solar irradiance. Under the clear-sky conditions of Day 1, the flux exhibits a parabolic profile, with a maximum of 16.6 kg/(m^2^h) at noon.

On Day 2, the flux remains below 2.1 kg/(m^2^h) for most of the day, except for a sharp rise at 10:00 when it temporarily increases to 10.8 kg/(m^2^h). Day 3, in contrast, shows a steady rise in flux up to 4.2 kg/(m^2^h) at 13:45, followed by a sudden jump to 6.3 kg/(m^2^h) at 15:00. Afterward, the flux declines rapidly to about 1 kg/(m^2^h) by the end of the day. Day 4 demonstrates the highest performance among all days with a peak of 18.4 kg/(m^2^h) at 13:45.

The average daily water flux is reported in Figure 13b. While Day 1 yields a reasonable average of 8.5 kg/(m^2^h), the highest average is achieved on Day 4 with 10.4 kg/(m^2^h). In contrast, Days 2 and 3 exhibit significantly lower averages of 2.0 and 2.8 kg/(m^2^h), respectively.

### 4.5. Performance Assessment of the Solar Multistage HF-WGMD System

In the following sections, the thermal performance and productivity of a multistage HF-WGMD desalination system powered by solar heating are examined under the weather conditions of Day 1. Particular attention is given to the effect of heat recovery through the module’s cooling channel on overall system performance.

In all cases, the desalination system is operated with a maximum feed water temperature of approximately 93 °C to ensure a consistent baseline across configurations. To achieve this inlet temperature, different total solar collector areas require different numbers of stages, owing to the heat recovery capability of the WGMD process. Specifically, smaller total collector areas necessitate a larger number of stages. For example, collector arrays with total areas of 10, 20 and 35 m^2^ require 40, 15 and 5 stages, respectively, while a collector area of 50 m^2^ requires only a single stage.

To justify these combinations, it is given that operating at a higher feed temperature improves the performance of the multistage HF-WGMD module. Through a direct iterative process, it was determined that 40 stages satisfy the temperature limit under the given conditions and 10 m^2^ collector array area. Increasing the number of stages would cause the feed temperature to exceed the allowable limit, while decreasing the number of stages would lower the feed temperature and reduce system performance. Therefore, for the given conditions and a collector array area of 10 m^2^, the optimal number of stages is 40. The same approach was applied to determine the appropriate number of stages for collector array areas of 20, 35, and 50 m^2^. This behavior with different number of stages arises because increasing the stages allows the cooling stream (saline water) to progressively recover heat lost through evaporation and thermal conduction. As a result, the saline water enters the solar collector at a higher temperature, thereby reducing the thermal load on the collector and enabling smaller collector array sizes to achieve the same feed water inlet temperature.

All collector arrays are designed with a fixed total width of 5 m, corresponding to five parallel rows, while the number of series rows is varied to be 2, 4, 7 and 10, yielding total collector areas of 10, 20, 35 and 50 m^2^, respectively. Each row consists of 40 solar tubes.

#### 4.5.1. Thermal Performance

The thermal performance of the multistage HF-WGMD desalination systems is evaluated in terms of the contributions of heat recovery and solar heating to feed water preheating and the system’s specific thermal energy consumption.

**Figure 14:**
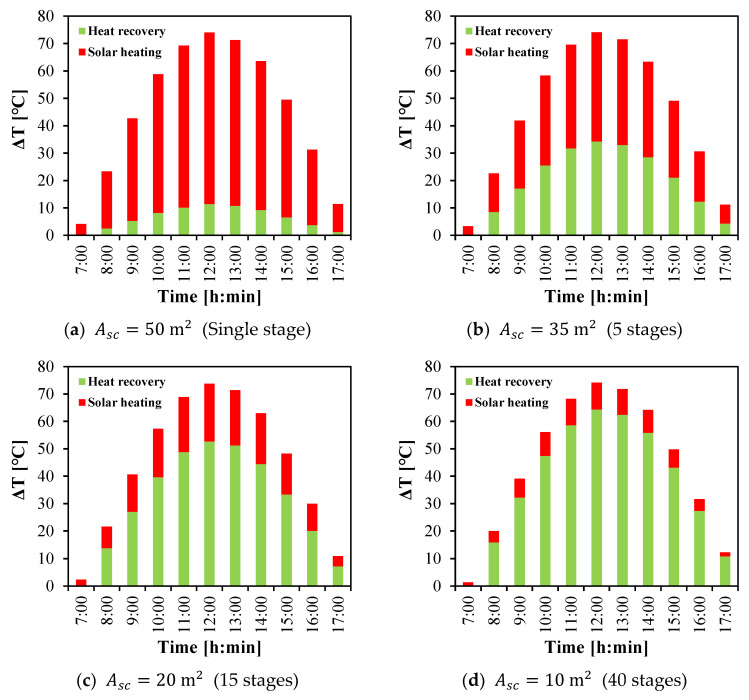
Temperature rise in saline water due to cooling water heat recovery and solar heating in multistage HF-WGMD system integrated with FPC array.

The temperature rise in the saline water as expressed by $\left(T_{fi}-T_{ci}\right)$ or ∆T is achieved by the combined effect of heat recovery and solar heating as shown in Figure 2. Figure 14 shows this temperature rise under different study cases. A comparable temperature rise profile across the different study cases is obtained due to using the same solar source with the almost same upper temperature limit and the same coolant temperature.

For all cases, the heat recovery contribution follows a parabolic trend similar to the solar irradiance of Day 1. As solar intensity increases, feed water temperature rises, leading to higher evaporation and conduction losses that are subsequently recovered by the cooling stream. Consequently, the dependence on solar heating decreases with increasing number of stages. For instance, with a 50 m^2^ collector array and a single HF-WGMD stage, the heat recovery effect increases from zero in the morning to 11.5 °C at noon, while an additional 62.6 °C is provided by solar heating (Figure 14a). Incorporating five stages with a 35 m^2^ collector array increases the recovery effect to 34.3 °C at noon, reducing the solar heating requirement by 36.4% (Figure 14b). Extending the configuration to 15 stages ($A_{sc}=20\;m^{2}$) and 40 stages ($A_{sc}=10\;m^{2}$) further enhances recovery to 52.7 °C and 64.4 °C, respectively, corresponding to 66.3% and 84.3% savings in solar heating (Figure 14c,d).

**Figure 15:**
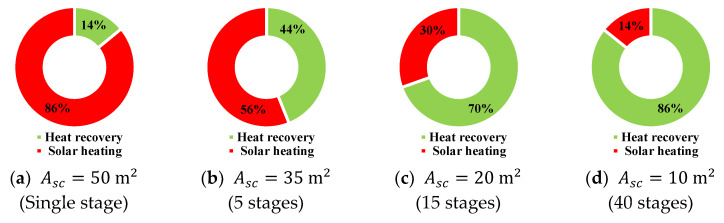
Cooling water heat recovery and solar heating proportions of total heat energy supplied to saline water fed to multistage HF-WGMD system integrated with FPC array.

The relative contributions of heat recovery and solar heating are summarized in Figure 15. For a single-stage configuration, solar heating supplies up to 86% of the required energy, while only 14% is covered by heat recovery (Figure 15a). Increasing the number of stages to 5 and 15 significantly shifts this balance, with heat recovery providing 44% and 70% of the total heating, respectively (Figure 15b,c). The 40-stage system demonstrates the most effective utilization of heat recovery, supplying 86% of the required heating with only 14% supplied by solar input (Figure 15d).

**Figure 16:**
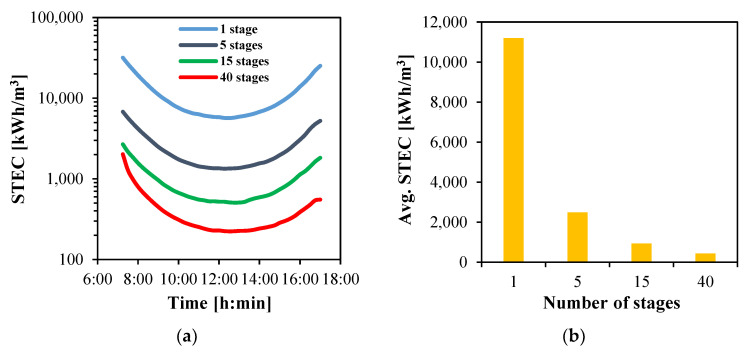
The STEC of multistage HF-WGMD system integrated with FPC array; (**a**) instantaneous and (**b**) average.

The instantaneous STEC of the solar heating unit is presented in Figure 16a. In all cases, STEC decreases during the morning, reaching its minimum around noon when productivity is highest, and then increases again towards evening. Systems with multiple stages show drastic reductions in STEC. At 12:30, values decline to 1343, 511 and 223 kWh/m^3^ for systems with 5, 15 and 40 stages, respectively, corresponding to energy savings of 76.3%, 91.0% and 96.1% relative to the single-stage case.

The average STEC values, reported in Figure 16b, confirm this trend. The single-stage system consumes 11,192 kWh/m^3^, whereas the consumption decreases to 2488 and 931 kWh/m^3^ for the 5- and 15-stage systems, respectively. The lowest energy consumption is observed with 40 stages, where the average STEC drops to only 424 kWh/m^3^.

#### 4.5.2. Overall Efficiency

**Figure 17:**
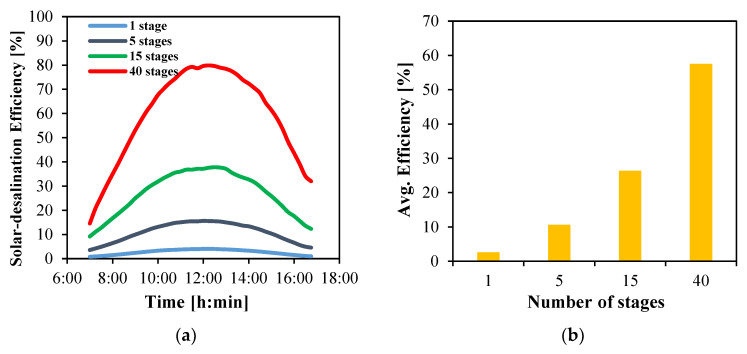
Solar desalination efficiency of multistage HF-WGMD system integrated with FPC array; (**a**) instantaneous and (**b**) average.

The efficiency of the solar desalination system is evaluated for the four multistage configurations. The instantaneous efficiency profiles are shown in Figure 17a. The use of a higher number of stages consistently enhances system efficiency, primarily due to the smaller collector array sizes required. For example, the 40-stage system has a peak of 79.9%, while the 15-stage and 5-stage systems yield significantly lower peak efficiencies of 37.6% and 15.5%, respectively. The lowest performance is observed for the single-stage system, which reaches only a maximum of 4.1%.

The average efficiencies over the daytime are presented in Figure 17b. The 40-stage system achieves a reasonable average efficiency of 57.5%, while the 15- and 5-stage systems record 26.4% and 10.7%, respectively. The single-stage system shows a dramatic decline, utilizing only 2.6% of the total solar energy absorbed by the 50 m^2^ collector.

#### 4.5.3. Productivity

**Figure 18:**
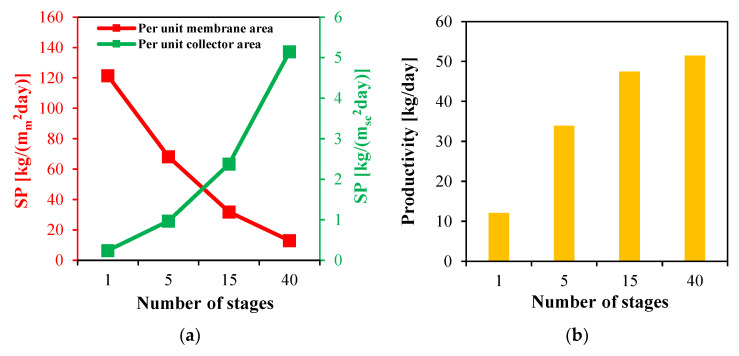
(**a**) Specific productivity per unit membrane area and per unit solar collector area and (**b**) productivity of multistage HF-WGMD system integrated with FPC array.

The productivity of the multistage HF-WGMD desalination systems is evaluated on two bases: per unit membrane area and per unit collector area, as shown in Figure 18a.

On a membrane-area basis, specific productivity decreases with increasing number of stages due to stronger temperature and concentration polarization effects along the extended module length. The single-stage system achieves the highest SP, producing 121.5 kg/m_m_^2^day. However, the 40-stage configuration yields the lowest value of 12.9 kg/(m_m_^2^day), representing an 89.4% reduction relative to the single-stage system.

In contrast, productivity per unit collector area follows an opposite trend. As the number of stages increases, the required collector area decreases, while the extended module length enhances overall production. Consequently, the single-stage system achieves only 0.2 kg/(m_sc_^2^day) of freshwater, whereas values increase markedly to 1, 2.4 and 5.1 kg/(m_sc_^2^day) for the 5-, 15- and 40-stage systems, respectively. These represent specific productivity enhancements of approximately 5, 12 and 25.5 times compared to the single-stage system.

The overall system productivity is presented in Figure 18b. The single-stage system produces 12.1 kg/day, while the 5-, 15-, and 40-stage systems achieve 33.9, 47.5 and 51.5 kg/day, corresponding to 2.8-, 3.9- and 4.3-fold improvements, respectively.

It is important to note that these relatively modest production values result from the compactness of the HF-WGMD modules used in this study, each with a shell diameter of 50 mm and a length of 300 mm, occupying only 5.9 × 10^−4^ m^3^. Thus, future designs should consider arranging a number of such multistage HF-WGMD modules in parallel to achieve competitive production levels suitable for different applications. Moreover, the present results correspond to weather conditions in March in Alexandria, characterized by low to medium solar irradiance, which should be taken into account when evaluating the system’s performance under more favorable solar conditions.

It can be concluded that the configuration combining a 10 m^2^ collector array with 40 module stages achieved the best overall energy and desalination performance under the given conditions. Consequently, if a total collector area of 50 m^2^ were available under similar conditions, it should ideally be divided into five separate solar arrays (10 m^2^ each), each coupled with 40 HF-WGMD module stages, to achieve optimal performance while maintaining the feed temperature below the boiling limit.

From an investment perspective, the proposed solar-driven HF-WGMD system benefits from modular scalability and the use of commercially available flat-plate collectors, which can substantially reduce the capital cost compared to conventional solar desalination technologies. The estimated system cost is primarily governed by the solar collector area and membrane module configuration, suggesting that multi-stage arrangements with smaller collector areas can offer a favorable cost-to-yield ratio. Looking ahead, the integration of low-cost polymeric membranes, advanced heat recovery designs, and hybridization with photovoltaic–thermal (PV/T) systems could further enhance the system’s economic feasibility and energy efficiency. These features position the HF-WGMD concept as a promising candidate for decentralized and sustainable desalination applications in water-stressed, solar-rich regions.

## 5. Conclusions

A two-dimensional transient CFD model was developed to simulate a hollow fiber water gap membrane distillation (HF-WGMD) module integrated with a flat-plate solar collector system operating with 35000 ppm saline feed. The model was validated against experimental data, demonstrating strong agreement and confirming its reliability for performance prediction. It was subsequently applied to analyze the effects of collector design, operating parameters, weather variability, and multistage configurations on energy recovery, desalination efficiency, and freshwater productivity.

- The solar collector area strongly influences productivity; increasing from 10 m^2^ to 50 m^2^ enhances average water flux by a factor of 6.4, reaching 10.9 kg/(m^2^·h).
- Reducing the total collector width (≤5 m) improves performance.
- Lower working fluid flow rates slightly increase flux, from 10.5 to 12.1 kg/(m^2^·h) as flow decreases from 20.3 kg/min to 6.7 kg/min.
- Water flux follows diurnal solar variation, peaking at 18.4 kg/(m^2^·h) with a 35 m^2^ collector on Day 4.
- Multistage HF-WGMD configurations significantly reduce solar energy demand through internal heat recovery; a 40-stage system requires only 10 m^2^ of collectors (14% solar share) compared with 50 m^2^ for a single stage (86% solar share).
- The 40-stage system achieves a minimum instantaneous STEC of 223.4 kWh/m^3^ (at the midday) and an average of 424 kWh/m^3^, on Day one.
- Solar desalination efficiency improves from 2.6% (single-stage) to 57.5% (40-stage), with specific productivity per unit collector area increasing 25.5-fold to 51.5 kg/day of freshwater.

Overall, the study demonstrates that multistage solar-driven HF-WGMD systems offer substantial improvements in energy utilization and freshwater yield, underscoring their strong potential for scalable and sustainable desalination applications.

## Figures and Tables

**Table 1:** Geometrical and operating parameters of HF-WGMD module.

Parameter	Symbol	Value	Unit
Feed channel radius	$r_{f}$	0.4	mm
Hollow fiber outer radius	$r_{m}$	0.58	mm
Water gap outer radius	$r_{g}$	1.42	mm
Cooling tube outer radius	$r_{t}$	1.7	mm
Cooling channel outer radius	$r_{c}$	2.62	mm
Module effective length	*L*	300	mm
Number of fibers per module	$N_{f}$	91	-
Feed inlet salinity	-	35000	ppm
Feed water inlet velocity	$U_{fi}$	1.62	m/s
Feed water thermal conductivity	$k_{f}$	0.64	W/(m K)
Membrane thermal conductivity	$k_{m}$	0.07	W/(m K)
Membrane porosity	*ε*	82	%
Membrane pore tortuosity	τ	1.7	-
Membrane pore diameter	$d_{p}$	0.16	μm
Water gap salinity	-	0.0	ppm
Cooling tube thermal conductivity	$k_{t}$	0.445	W/(m K)
Cooling water inlet velocity	$U_{ci}$	0.065	m/s
Number of module stages	$N_{stages}$	1, 5, 15 & 40	-
Vapor molecular mass (H_2_O)	$M_{w}$	18	g/mol
Salt molecular mass (NaCl)	$M_{NaCl}$	58.5	g/mol

**Table 4:** Geometrical parameters of flat-plate solar collectors.

Parameter	Symbol	Value	Unit
Total collector array area	$A_{sc}$	10 to 50	m^2^
Total collector array width	$W_{sc}$	2 to 25	m
Single solar collector dimensions	-	1 × 1	m^2^
Number of solar tubes per row (in parallel)	$N_{st}$	20 to 100	-
Solar tube inner diameter	$d_{sti}$	9.4	mm
Solar tube outer diameter	$d_{sto}$	12.7	mm
Solar fluid convection heat transfer coefficient	$h_{sf}$	300	W/(m^2^K)
Overall heat loss coefficient	$U_{L}$	6	W/(m^2^K)
Absorber plate thermal conductivity	$k_{p}$	385	W/(m K)
Absorber plate thickness	$\delta_{p}$	0.4	mm
Working fluid inlet velocity	$U_{sfi}$	0.04	m/s
Glass transmissivity	$\tau_{g}$	0.9	-
Plate absorptivity	$\alpha_{p}$	0.95	-

**Table 5:** Boundary conditions of mass conservation equations for feed and membrane domains.

Domain	Position	Boundary Condition
Feed channel	r=0	$\frac{\partial c_{f}}{\partial r}=0$
	$r=r_{f}$	$-D_{w}\frac{\partial c_{f}}{\partial r}=-D_{m}\frac{\partial c_{m}}{\partial r}$
	z=0	$c_{f}=c_{fi}$
	z=L	$\frac{\partial c_{f}}{\partial z}=0$
Membrane	$r=r_{f}$	$c_{m}=c_{hot}$
	$r=r_{m}$	$c_{m}=c_{cold}$
	z=0	$\frac{\partial c_{m}}{\partial z}=0$
	z=L	$\frac{\partial c_{m}}{\partial z}=0$

**Table 6:** Boundary conditions of momentum conservation equations for feed and coolant domains.

Domain	Position	Boundary Condition
Feed channel	r=0	$\frac{\partial u_{f}}{\partial r}=0,\;\frac{\partial w_{f}}{\partial r}=0$
	$r=r_{f}$	$u_{f}=0,w_{f}=0$
	z=0	$u_{f}=0,w_{f}=U_{fi}$
	z=L	$P_{f}=P_{atm}$
Cooling channel	$r=r_{t}$	$u_{c}=0,w_{c}=0$
	$r=r_{c}$	$\frac{\partial u_{c}}{\partial r}=0,\frac{\partial w_{c}}{\partial r}=0$
	z=0	$P_{c}=P_{atm}$
	z=L	$u_{c}=0,w_{c}={-U}_{ci}$

**Table 7:** Boundary conditions of energy conservation equations for HF-WGMD module domains.

Domain	Position	Boundary Condition
Feed channel	r=0	$\frac{\partial T_{f}}{\partial r}=0$
	z=0	$T_{f}=T_{fi}$
	z=L	$\frac{\partial T_{f}}{\partial z}=0$
Membrane	z=0	$\frac{\partial T_{m}}{\partial z}=0$
	z=L	$\frac{\partial T_{m}}{\partial z}=0$
Water gap	z=0	$\frac{\partial T_{g}}{\partial z}=0$
	z=L	$\frac{\partial T_{g}}{\partial z}=0$
Cooling tube	z=0	$\frac{\partial T_{t}}{\partial z}=0$
	z=L	$\frac{\partial T_{t}}{\partial z}=0$
Cooling channel	$r=r_{c}$	$\frac{\partial T_{c}}{\partial r}=0$
	z=0	$\frac{\partial T_{c}}{\partial z}=0$
	z=L	$T_{c}=T_{ci}$

**Table 8:** Average feed inlet temperature of HF-WGMD module integrated with different solar collectors’ areas during the operation on Day 1.

Time	Average Feed Inlet Temperature [℃]			
	$A_{sc}=10\;m^{2}$	$A_{sc}=20\;m^{2}$	$A_{sc}=35\;m^{2}$	$A_{sc}=50\;m^{2}$
8:00	24.1	29.4	34.7	38.1
12:00	44.8	61.4	78.9	88.9
15:00	36	47.4	58.6	65.7

**Table 9:** Average feed–membrane interface temperature during daytime operation of four days in March 2025.

Time	Average Feed–Membrane Interface Temperature [℃]			
	Day 1	Day 2	Day 3	Day 4
10:00	61.6	64.6	35	69.5
12:15	73.2	41.8	44.8	74.3
13:45	67.6	41.1	46.1	79.1
15:00	55.7	37	52.6	67.9

## Data Availability

The original contributions presented in this study are included in the article. Further inquiries can be directed to the corresponding author.

## Abbreviations

A	Area [m^2^]
$a_{w}$	Water activity coefficient
$C_{p}$	Specific heat at constant pressure [J/(kg K)]
c	Concentration [mol/m^3^]
D	Diffusion coefficient [m^2^/s]
$D_{Kn}$	Knudsen diffusion coefficient [m^2^/s]
$D_{Or}$	Ordinary molecular diffusion coefficient [m^2^/s]
d	Diameter [m]
$d_{p}$	Pore diameter [m]
$d_{sti}$	Solar tube inner diameter [m]
$d_{sto}$	Solar tube outer diameter [m]
F	Standard fin efficiency
$F_{R}$	Heat removal factor
$F^{\prime }$	Collector efficiency factor
$h_{fg}$	Latent heat of vaporization [J/kg]
$h_{sf}$	Solar fluid convection heat transfer coefficient [W/(m^2^K)]
J	Water flux [kg/m^2^h]
Kn	Knudsen number
k	Thermal conductivity [W/(m K)]
L	Module length [m]
M	Molecular mass [g/mol]
$\dot{m}$	Mass flow rate [kg/s]
$N_{f}$	Number of fibers per module
$N_{st}$	Number of solar tubes
$N_{stages}$	Number of module stages
P	Pressure [Pa]
ppm	Concentration in parts per millions
$Q_{u}$	Useful heat gain [W]
$q_{Latent}$	Absorbed/expelled heat flux [W/m^2^]
r	Radius [mm]
$\bar{R}$	Universal gas constant [J/(mol K)]
$S_{i}$	Solar irradiance [W/m^2^]
$S_{st}$	Solar tube spacing [m]
SP	Specific productivity [kg/(m^2^day)]
STEC	Specific thermal energy consumption [kWh/m^3^]
T	Temperature [℃]
t	Time [h]
*U*	Flow stream mean velocity [m/s]
$U_{L}$	Overall heat loss coefficient of FPC [W/(m^2^K)]
u	Velocity component in radial direction [m/s]
v	Specific volume [m^3^/kg]
W	Width [m]
$W_{v}$	Vapor content [kg_v_/kg_a_]
w	Velocity component in axial direction [m/s]
$x_{NaCl}$	Salt mole fraction
$x_{w}$	Water mole fraction
Subscripts	
a	Air
atm	Atmospheric
c	Coolant
f	Feed
g	Water gap
i	Inlet
m	Membrane
o	Outlet
p	Absorber plate
r	Radial direction
sc	Solar collector
sf	Solar working fluid
st	Solar tube
sat	Saturation
t	Cooling tube
v	Vapor
w	Water
z	Axial direction
Greek symbols	
$\alpha_{p}$	Plate absorptivity
$\delta_{p}$	Absorber plate thickness
*ε*	Membrane porosity
η	Solar desalination efficiency
*μ*	Fluid dynamic viscosity [Pa s]
ρ	Fluid density [kg/m^3^]
σ	Molecule collision diameter [m]
τ	Membrane tortuosity
$\tau_{g}$	Solar collector glass transmissivity
